# Role of Cytokines and Other Soluble Factors in Tumor Development: Rationale for New Therapeutic Strategies

**DOI:** 10.3390/cells12212532

**Published:** 2023-10-27

**Authors:** Andrea Cavazzoni, Graziana Digiacomo

**Affiliations:** Department of Medicine and Surgery, University of Parma, 43126 Parma, Italy; andrea.cavazzoni@unipr.it

Many cytokines control tumor development by directly lowering cancer cell proliferation and inducing apoptotic cell death, or indirectly by activating the antitumoral activity of specific immune cells such as NK or CD8^+^ T-lymphocytes. These cytokines exert their functions in the tumor stroma compartment known as the tumor microenvironment (TME); in this region, an important regulation of tumor growth is exerted by specific cytokines that can also stimulate tumor angiogenesis.

Tumor and stromal cells present in the TME (tumor-infiltrating lymphocytes (TILs), cancer-associated fibroblasts (CAFs), cancer-associated macrophages, and endothelial cells) secrete high levels of pro-angiogenic factors that can create an abnormal vasculature, resulting in poorly perfused tumors or several inflammatory mediators, such as TNF-α, IL-6, TGF-β, and IL-10, which are involved in both the initiation and progression of cancer. 

The Special Issue entitled “Role of Cytokines and Other Soluble Factors in Tumor Development: Rationale for New Therapeutic Strategies” focuses on the roles of some cytokines as mediators of tumor progression and on the activities of novel therapeutic approaches based on the employment of cytokines and derivatives as a single agent or coupled with immune checkpoint inhibitors to fight TME.

The Special Issue published ten articles consisting of one original article, eight review articles, and one systematic review.

It is known that chronic inflammation is implicated in the development and progression of several cancers. The epithelial–mesenchymal transition (EMT) is a process connected to cancer progression and metastasis. Inflammatory mediators, such as cytokines and other soluble factors, oxidative stress, or hypoxia can promote the acquisition of EMT-like features in cancer cells. 

Ray et al. [1] evaluated the effect of cytokines on EMT in gynecological cancers and their possible therapeutic implications. By employing specific keywords in several databases and seventy-one articles, the authors found that various cytokines, such as TGF-β, TNF-α, and IL-6, promoted EMT changes in ovarian, cervical, and endometrial cancers. In particular, the EMT variations observed were from epithelial to mesenchymal morphologies, downregulation of the epithelial markers E-cadherin/β-catenin, upregulation of the mesenchymal markers N-cadherin/vimentin/fibronectin, and upregulation of the EMT-transformation factors (EMT-TF) SNAI1/SNAI2/TWIST/ZEB. Considering that cytokines can induce EMT-promoting gynecological cancer development and metastasis, the authors hypothesized that the development of new therapeutic strategies that target the cytokines or their signaling pathways in the EMT could be interesting in preventing cancer progression and drug resistance.

The review by Balsano et al. [2] described the role of the TGF-β pathway and other molecules in cancer onset and progression with a focus on their involvement in cachexia. Cachexia is a multifactorial metabolic and immune system imbalance that results in a loss of muscle mass and function with a severe impact on the quality of life and survival of cancer patients. IL-6, TNF-α, TGF-β, and MIC-1/GDF15, a member of the TGF-β superfamily, are known as drivers of cachexia. TGF-β causes muscle loss through myostatin-based signaling, involved in the reduction in protein synthesis and enhanced protein degradation. Moreover, TGF-β induces inhibin and activin, causing weight loss and muscle depletion, whereas MIC-1/GDF15 leads to anorexia and thus, albeit indirectly, to muscle wasting, acting on the hypothalamus’ center. The authors suggest the validation of novel biomarkers to identify patients with a risk of cachexia and a potential TGF-β-inhibiting therapy to prevent tumor progression and avoid cachexia in cancer.

The review by Raskova et al. [3] summarized the role of IL-6 signaling pathways in cancer biology. IL-6 has been identified as a cytokine abundantly present in the TME of various tumor types. IL-6 not only plays a significant role during tumorigenesis, but it also facilitates a series of events that must occur in the formation of metastasis. The authors summarized that targeting principal components of IL-6 signaling is an intensively studied approach in preclinical cancer research, especially in metastasis suppression. However, IL-6 signaling is not an isolated phenomenon. The authors suggested that a possible therapeutic approach could be the combination with inhibitors of other signaling pathways, based on tumor type characteristics. 

The review of Braumüller et al. [4] focused its attention on the cytokine networks between cancer cells and the TME in colorectal cancer (CRC) and the possible therapeutic strategies. In CRC, the presence of specific TILs can be associated with a good or poor prognosis; the infiltration of TH1 cells is associated with a good prognosis, and, otherwise, a poor prognosis is linked to TH17 cells that secrete substantial amounts of interleukin-17 (IL-17) involved in chronic inflammation. Soluble inflammatory factors, specifically cytokines and chemokines, exert promotion or suppressive tumor expansion. In CRC, different cytokines have a pro-tumorigenic effect; some of these, such as IL-6, IL-1a, and IL-1b, are increased in CRC. On the contrary, IL-18 is decreased in CRC patients, indicating a significant anti-tumorigenic effect. Cytokines have been known for their antitumoral efficacy for decades, and they are possible candidates for developing new drugs. In conclusion, the authors suggested that different cytokine pools in the TME influence tumor progression and therapeutic strategies. For this purpose, the authors suggested the combination of cytokines with antitumor activities with immunotherapy, chemotherapy, or radiotherapy.

Another interleukin involved in different tumors is IL-33, as reported by Pisani et al. [5] IL-33 is produced by various cell types, such as stromal cells, including fibroblasts, cancer-associated fibroblasts (CAF), and mesenchymal stromal cells, and it is involved in angiogenesis and cancer progression. The role of IL-33 in tumorigenesis was first identified in breast cancer. In this review, the authors showed that the IL-33/ST2 axis plays a complex role in the carcinogenesis of gastrointestinal tract tumors. The pro- or antitumorigenic effects of IL-33 are not clear, probably depending on the specific cancer type and the microenvironment. The authors summarized the recent advances in gastrointestinal tract cancers, and further studies are needed to better understand how IL-33 can be targeted for potential therapeutic strategies in the context of gastric cancer.

Many cell populations present in the TME are involved in tumor progression and exhaustion of T-cell activity, making immunotherapy approaches less effective in some solid tumors. Recently, the development of new cellular strategies based on the employment of CAR-T cells has been very successful in hematologic malignancies, with the approval of six FDA CAR-T cell therapies. Nevertheless, the success in these tumor types and the extension of this approach to solid tumors encountered some problems connected to the immune-suppressive properties of the TME. The review proposed by Cheever et al. [6] discussed the mechanisms adopted by TME to reduce the antitumor properties of CAR-T cells, in particular the homing and the difficulties of physically entering the neoplastic tissue. Moreover, the lack of oxygen and nutrients in the TME is responsible for the exhaustion of CAR-T cells. Finally, antigen loss has been documented as another mechanism of tumor escape, often associated with the expression of PD-L1 on the tumor cells. With the aim to circumvent these aspects, the review highlighted new experimental approaches such as the combination with immunotherapy, the structural modification of CAR-T cell receptor, and, finally, the addiction of oncolytic viruses to change from an immunosuppressive to an inflammatory TME phenotype.

In the extremely high heterogeneous composition of TME in terms of cell populations, the tumor-infiltrating immune cells (TIIC) play an important function as the main responsible for the failure of immunotherapy; moreover, an increasing number of studies have shown that these cells play an important role in the metastasization process. In the review from Li et al. [7], it is reported that NK cells, M2 macrophages, and dendritic cells (DC), by secreting cytokines, are responsible for the induction of the EMT, one of the critical processes that promote cancer progression and, finally, metastasis. In addition, the tumor-associated macrophages and CD4^+^ T-cells are involved in the degradation of the extracellular matrix, and macrophages and NK cells are directly involved in the regulation of tumor angiogenesis because of NK cell reprogramming. Finally, a lot of data reported that immune cells are the main component of the pre-metastatic niche (PMN) in the host organ before tumor metastasis and can contribute to tumor spread and dissemination.

One of the most known cytokines is IL-2. From its discovery in 1976, a large amount of experimental data revealed its main function as a potent growth factor, promoting the expansion and maintenance of T-cell function (both CD4 and CD8) and NK cells, suggesting an important role as anticancer agent. Several clinical trials have been proposed, with the aim of elucidating this function, but, unfortunately, only in melanoma and renal cell carcinoma patients, the addiction to high doses of IL-2 produced a marginal effect. This is a consequence of the high toxicity observed after administration of IL-2; moreover, IL-2 is also responsible for T-reg activation, a population often present in the TME, as the main responsible for the failure of immunotherapy. For this reason, the review by Ko et al. [8] proposed new strategies to overcome these limitations. They reported the employment of new synthetic IL-2-fusion protein variants to increase the anti-cancer properties and simultaneously reduce the ability to stimulate immune T-reg cells in the TME, responsible for blocking cytotoxic T-cell activities. Some of these new compounds (ALKS 4230, BMS, SAR444245, and TransCon IL-2 β/γ) entered phase 1–3 clinical trials in monotherapy or were associated with immune checkpoint inhibitors. In 2021, the ALKS 4230 agent (nemvaleukin alfa) received FDA approval for the treatment of mucosal melanoma as a single agent. The clinical development of these variants with different mechanisms of action holds promise for significantly enhancing the therapeutic effect of IL-2 with more relevant activation of anti-tumor cytotoxic T-cells.

One of the most important cytokines involved in NK and CD8^+^ T-cell activation is IL-15. This cytokine is a member of 4-α-helical bundle family of cytokines, and its administration has been considered a valuable agent to activate immune response for tumor treatment. As a consequence of its short half-life (~2.5 h) and dose-limiting toxicities, the intravenous delivery of IL-15 failed to produce significant results. Lui et al. reported that a chimeric variant (N803) of IL-15 has been synthesized and tested for its activity to overcome these limitations. Several preclinical data enforced the role of N803, and, in particular, it produced a significant increase and activation of both NK and CD8^+^ T-cells, with a significant reduction in tumor mass in different cancer types. Further experimental evidence has been reported when N803 was combined with other agents, in particular, the immune checkpoint inhibitors, anti-histone-deacetylase compounds, and, finally, cancer vaccines targeting the pan-cancer carcinoembryonic antigen (Ad-CEA) or Twist1 (Ad-Twist1).

Growing evidence confirmed the use of N803 in combinatorial therapies, including those involved in the inhibition of suppressive phenotype often detected in the TME, with the intent to potentiate the effect of immune checkpoint inhibitors also in patients initially refractory to this kind of therapy [9]. 

In the research article by Matarrese et al. [10], the authors investigated how CAFs modulate a tumor cell’s phenotype by secreting some specific cytokines. The study was conducted in two NSCLC cell lines treated with a mix of four chemokines known to be secreted by CAF. A significant increase in cell motility, associated with an intracellular redistribution of mitochondria localization, has been reported. These morphological and functional alterations, indicative of the acquisition of a more aggressive cancer phenotype, have been linked to changes in the expression of EMT markers. Moreover, a reduction in the Yap protein has been reported in these experimental conditions. Yap signaling is mainly engaged in several intracellular processes; it controls the induction of the autophagic process, as in Yap-negative NSCLC (by siRNA approach), and the autophagic machinery is blocked. Globally, these results suggested that cytokines produced by CAFs are responsible for a dramatic switch of NSCLC cells toward more aggressive, mesenchymal features.

## References

[1] Ray I., Michael A., Meira L.B., Ellis P.E. (2023). The Role of Cytokines in Epithelial-Mesenchymal Transition in Gynaecological Cancers: A Systematic Review. Cells.

[2] Balsano R., Kruize Z., Lunardi M., Comandatore A., Barone M., Cavazzoni A., Re Cecconi A.D., Morelli L., Wilmink H., Tiseo M. (2022). Transforming Growth Factor-Beta Signaling in Cancer-Induced Cachexia: From Molecular Pathways to the Clinics. Cells.

[3] Raskova M., Lacina L., Kejik Z., Venhauerova A., Skalickova M., Kolar M., Jakubek M., Rosel D., Smetana K., Brabek J. (2022). The Role of IL-6 in Cancer Cell Invasiveness and Metastasis-Overview and Therapeutic Opportunities. Cells.

[4] Braumuller H., Mauerer B., Andris J., Berlin C., Wieder T., Kesselring R. (2022). The Cytokine Network in Colorectal Cancer: Implications for New Treatment Strategies. Cells.

[5] Pisani L.F., Teani I., Vecchi M., Pastorelli L. (2023). Interleukin-33: Friend or Foe in Gastrointestinal Tract Cancers?. Cells.

[6] Johnson A., Townsend M., O’Neill K. (2022). Tumor Microenvironment Immunosuppression: A Roadblock to CAR T-Cell Advancement in Solid Tumors. Cells.

[7] Li H.X., Wang S.Q., Lian Z.X., Deng S.L., Yu K. (2022). Relationship between Tumor Infiltrating Immune Cells and Tumor Metastasis and Its Prognostic Value in Cancer. Cells.

[8] Ko B., Takebe N., Andrews O., Makena M.R., Chen A.P. (2023). Rethinking Oncologic Treatment Strategies with Interleukin-2. Cells.

[9] Lui G., Minnar C.M., Soon-Shiong P., Schlom J., Gameiro S.R. (2023). Exploiting an Interleukin-15 Heterodimeric Agonist (N803) for Effective Immunotherapy of Solid Malignancies. Cells.

[10] Matarrese P., Vona R., Ascione B., Cittadini C., Tocci A., Mileo A.M. (2023). Tumor Microenvironmental Cytokines Drive NSCLC Cell Aggressiveness and Drug-Resistance via YAP-Mediated Autophagy. Cells.

